# Hyponatremia in the intensive care unit: How to avoid a Zugzwang situation?

**DOI:** 10.1186/s13613-015-0066-8

**Published:** 2015-11-09

**Authors:** Cédric Rafat, Martin Flamant, Stéphane Gaudry, Emmanuelle Vidal-Petiot, Jean-Damien Ricard, Didier Dreyfuss

**Affiliations:** AP-HP, Service de Réanimation Médico-Chirurgicale, Hôpital Louis Mourier, Colombes, France; AP-HP, Urgences Néphrologiques et Transplantation Rénale, Hôpital Tenon, Paris, France; AP-HP, Service de Physiologie Rénale, Hôpital Bichat, Paris, France; Université Paris Diderot, Sorbonne Paris Cité, Paris, France; INSERM, U1149, Centre de Recherche sur l’Inflammation, Paris, France; ECEVE UMR 1123, ECEVE, Paris, France; INSERM UMR 1137, IAME, Paris, France

**Keywords:** Hyponatremia, Hyponatremic encephalopathy, Osmotic demyelination, Central pontine myelinolysis, Arginine vasopressin, Extracellular fluid volume

## Abstract

Hyponatremia is a common electrolyte derangement in the setting of the intensive care unit. Life-threatening neurological complications may arise not only in case of a severe (<120 mmol/L) and acute fall of plasma sodium levels, but may also stem from overly rapid correction of hyponatremia. Additionally, even mild hyponatremia carries a poor short-term and long-term prognosis across a wide range of conditions. Its multifaceted and intricate physiopathology may seem deterring at first glance, yet a careful multi-step diagnostic approach may easily unravel the underlying mechanisms and enable physicians to adopt the adequate measures at the patient’s bedside. Unless hyponatremia is associated with obvious extracellular fluid volume increase such as in heart failure or cirrhosis, hypertonic saline therapy is the cornerstone of the therapeutic of profound or severely symptomatic hyponatremia. When overcorrection of hyponatremia occurs, recent data indicate that re-lowering of plasma sodium levels through the infusion of hypotonic fluids and the cautious use of desmopressin acetate represent a reasonable strategy. New therapeutic options have recently emerged, foremost among these being vaptans, but their use in the setting of the intensive care unit remains to be clarified.

## Background

Hyponatremia is the single most frequent electrolyte disturbance encountered in the intensive care unit (ICU) affecting as many as 24.5 % of the patients, depending on its biochemical definition [1]. Indeed, critically ill patients often cumulate multiple prerequisite factors rendering them susceptible to hyponatremia, namely impaired free water excretion, frequent administration of hypotonic fluids, and multiple morbid and drug-related conditions known to predispose to hyponatremia [1–3].

Symptoms associated with hyponatremia encompass a broad spectrum of clinical presentations ranging from subtle cognitive deficiencies to life-threatening neurological impairment including status epilepticus and brain herniation.

Furthermore, hyponatremia is known to be closely associated with an altered prognosis among critically ill patients [4–6] and this holds true even in the face of mildly decreased levels of sodium plasma (PNa) levels.

Not only does hyponatremia represent a serious threat for patients but its management also represents a great challenge for clinicians. In the setting of profound, symptomatic and acute hyponatremia, failure to timely correct, at least in part, hyponatremia places the patient at risk of hyponatremic encephalopathy, seizure and brain herniation whereas an overly rapid increase in plasma sodium concentration renders patient vulnerable to the much dreaded osmotic demyelination syndrome (OD) [7].

Herein, in this first part of a review on hyponatremia in the setting of the intensive care unit, we examine the key issues related to physiological principles, clinical manifestations of hyponatremia as well as the physiopathology of OD and its clinical aspects.

## Basic principles of sodium and water equilibrium

Although an extensive description of the physiopathology is beyond the scope of this study, a few principles ought to be kept in mind when examining the pathophysiology of hyponatremia. They can be summed up as follows: (1) the vast majority of cell membranes are permeable to water, with the notable exception of neurons. (2) In contrast, ions and other solutes (except urea in most clinical conditions) may not freely cross cell membranes, they are termed effective osmolytes. (3) Therefore these effective osmolytes contribute to tonicity which is the osmotic gradient relative to the differential concentrations of osmolytes across cell membranes. (4) Water moves across cell membranes in order to achieve an osmotic equilibrium, in other words the osmotic gradient across cell membranes dictates the distribution of water between the intracellular and extracellular compartments. (5) At the point of equilibrium, intracellular and extracellular osmolality are identical. (6) Given that the total osmolar content of the intracellular compartment is twice as high as that of the extracellular compartment, the total body water is divided accordingly. (7) Electrolytes and water disturbances, whether they concern the intra- or extracellular compartment, first occur via alterations of the extracellular compartment, which is located at the interface with the external environment. (8) Under physiological conditions, plasma osmolality is strictly regulated so that changes in sodium balance translate into variations of the extracellular volume. (9) Conversely, intracellular osmolarity is preponderantly determined by water balance. (10) At variance with extracellular compartment where the predominant electrolytes are sodium and chloride, the intracellular compartment is composed chiefly of organic osmolytes. (11) Hence, the intracellular osmotic content is slow to adapt when the sodium and water content of the extracellular compartment is disturbed. (12) This also implies that extracellular hypo- or hyperosmolality will result in increased or decreased cell volume, respectively. (13) Water and sodium balances are regulated through differential pathways. (14) Arginine vasopressin (AVP) is the key determinant of water balance and allows for a tight control of plasma osmolality, as any large and sustained variations of osmolality would otherwise compromise the cell volume, integrity and functions [8, 9]. (15) Plasma sodium is the preponderant cation in the extracellular compartment whereas potassium predominates in the intracellular compartment. (16) As predicted by Edelman, plasma sodium concentration is a function not only of total exchangeable sodium and total body water but also of total exchangeable potassium, as follows [10]:

$$ {\text{PNa}} = \frac{{1.11 \times {\text{Nae}} \times {\text{Ke}}}}{{{\text{TBW }}({\text{L}})}} - 25.6 $$

where PNa stands for plasma sodium concentration, Nae total exchangeable sodium and Ke total exchangeable potassium. (17) Consequently, a noticeable decrease in the total potassium body content will induce a decrease in plasma sodium concentration.

## Osmoregulation and key regulators of the sodium and water equilibrium

### Osmosensors: collecting the osmotic stimuli

Whenever plasma osmolality undergoes variations exceeding 1–3 % of its pre-set value (~290 mOsm/kg H_2_0), homeostatic mechanisms are unleashed in an endeavor to restore its normal value [11]. The nature and intimate cell structure of these “osmosensors” remain elusive. Nevertheless, it is believed that these osmotic receptors act as neurons and are thus capable of converting plasma osmolality variations into electric signals [12]. Central osmoreceptors are the key players in this dual process of osmolality sensing and transduction. They are found in different areas of the central nervous system (CNS) including the organum vasculosum laminae terminalis (OVLT), which serves as the primary central osmoreceptors, but their presence has also been suggested in the supraoptic nucleus (SON) and the subfornical organ [13]. The signal stemming from these peripheral osmoreceptors is then routed to the CNS through the vagus nerve. These structures transduce an osmotic stimulus into a nervous signal via transient receptor potential vanilloid 1 and 4 (TRPV1 and 4) proteins which constitute a large cation channel family. Cellular shrinkage caused by hyperosmolality triggers the opening of these channels which in turn causes the depolarization of these neurons [14]. A fine illustration of the pivotal involvement of TRPV channels in the osmoregulatory process is provided by animal models with impaired expression of TRPV1 in which case they display chronic hyperosmolarity despite having free access to fluids [15]. Similarly, *trpv4*−*/*− mice display defective osmoregulation when subjected to an osmotic challenge [16]. Aside from these central receptors, fluctuation in plasma osmolality is also captured by osmotic sensors disseminated along the digestive tract and its blood vessels both in humans and other mammals [17, 18]. For a detailed review in a central mechanisms of osmosensation and systemic osmoregulation, please refer to [12], figure 3.

Once the CNS has collected and integrated the data stemming from peripheral and central osmotic receptors, it elicits compensatory responses through multiple effectors, namely: AVP release, thirst and salt appetite regulation, and natriuresis.

### Arginine vasopressin: regulating water excretion

#### Arginine vasopressin: general considerations

Arginine vasopressin, a 9-amino-acid cyclic peptide, is synthesized in the SON and paraventricular nuclei (PVN) magnocellular neurons, yet it is transported by axons and both stored and released by the neurohypophysis. The two key determinants of AVP synthesis and secretion are osmotic stimuli and effective blood volume variations. Indeed, the pattern of AVP release is well articulated within the network of osmoreceptors. In fact, the magnocellular neurons receive afferences from the neurons located in the OVLT and SON [19, 20] as well as peripheral osmoreceptors [17]. Taurine, one of the organic osmolyte excreted by glial cells as part of the “regulatory volume decrease” also acts as a neurohumoral mediator by dampening the release of AVP in the setting of hypoosmolality [21]. Finally, magnocellular neurons themselves may also display osmoreceptive properties in a similar fashion to neurons of the OVLT and the SON [22].

The mechanism whereby AVP regulates water balance is by increasing the renal collecting ducts’ permeability to water. Indeed, once it has been released in the systemic circulation, AVP binds to the V2 receptor, a 7-transmembrane domain protein coupled to G-proteins which is located on the basolateral surface of the collecting ducts. The ensuing intratubular cascade of events involves AMPc/PKA dependent pathways and culminates in the expression of aquaporin-2 (AQP2) water channels in the luminal surface of the collecting ducts, whereas they lay dormant niched in intratubular vesicles in the absence of AVP stimulation. AQP2 thus serves as the regulated water entry route inside the tubule, as opposed to the exit route which depends upon constitutively expressed water channels (AQP3 and AQP4) found in the basolateral membrane of the tubules. However, the passive flux of water from the lumen of the tubule back to the systemic circulation is conditioned by the existence of an osmotic gradient between the tubular lumen (low) and the kidney’s interstitium (high), itself rendered possible by the corticopapillary gradient. [23]. AVP plays a vital part in sustaining this osmotic drive by enhancing the number of sodium channels (ENac), thus promoting sodium reabsorption in the cortical and outer medulla [24, 25]. For a comprehensive review and schematic representations of the interactions between AVP and the collecting duct, please refer to [26], figures 4 and 5.

#### Arginine vasopressin: osmotic regulation

Increase in effective plasma osmolality (which equates hypernatremia in most instances) stimulates AVP release in a linear fashion. On the contrary, decrease of effective osmolality (hypoosmotic hyponatremia) decreases AVP release [27]. As a result from AVP variation, values of urine osmolality may span from 50 to 1200 mOsm/kg of water in young and healthy subjects with an average value of 600 mOsm/kg of water [28]. Variations of plasma osmolality as small as 1–2 % induce noticeable variations of plasma AVP values which in turn promote significant variations of urine osmolality [29]. AVP is thus instrumental in the fine-tuning of water-free excretion in response to changes in plasma osmolality. Furthermore, the short half-life of AVP allows for a swift adaptation of the osmoregulatory system on a minute-to-minute basis [27].

#### The role of hemodynamic and cardiovascular stimuli on arginine vasopressin release

Equally as important as osmotic stimuli, effective arterial blood volume is a potent regulator of AVP release. In fact, arterial underfilling even prevails over osmotic stimuli, in case of competing and antagonist effects on AVP secretion. Whenever arterial blood underfilling is encountered, in such opposite circumstances as “true hypovolemia” or so called “effective hypovolemia” a clinical picture commonly found in decompensated cirrhosis and congestive heart failure, AVP release is elicited, even if PNa is decreased. In these clinical settings, patients with “effective hypovolemia” typically exhibit both neurohormonal patterns of arterial underfilling and an increased extracellular fluid volume status (ECFV) with interstitial edema and expanded total plasma and blood volume, in contrast with patients with “true hypovolemia”. The corresponding clinical picture is one of non- maximally diluted urine and an excessive water load will inevitably give rise to hyponatremia.

This is thanks to carotid, aortic and atrial baroreceptors which sense arterial unloading and relay the stimulus to the PVN and the SON via neural afferents alongside the vagus and glossopharyngeal nerves and the nucleus of the solitary tract. The result encompasses a wide selection of cardiovascular and hemodynamic effects mediated by the V1a receptor [30]. The vasopressor effect is indeed one of the best known actions of AVP and is a prominent component of compensatory mechanisms in case of severe hypovolemia (true or effective). It is rendered possible by the presence of V1a receptors in smooth muscle cells, over a broad range of vascular beds. In addition, AVP enhances the sensitivity of the baroreflex arch, perhaps though V1 receptors located in the area postrema [31] and may reinforce the sympathetic activity [32]. Other relevant actions of AVP on blood pressure control are indirectly mediated by the stimulation of mineralocorticoid [33] and glucocorticoid functions [30].

### Regulation of thirst

Plasma osmolality is a powerful determinant of thirst as a rise of as few as 5–10 mOsm/kg is sufficient to induce thirst [34]. Neurofunctional imaging has provided insights on the areas of the CNS which appear to be associated with the consciousness of thirst, the anterior cingulate and the insula being the foremost of these [35]. The subfornical organ and the OVLT, in addition to their osmoreceptive attribute, also integrate inputs deriving from peripheral receptors and systemic factors [36]. Among the latter, angiotensin II should be noted for being a remarkable dipsogenic stimulus [37] and is elevated in conditions of both “true” and “effective hypovolemia”.

### Salt appetite

Compared to thirst which manifests itself within 1–2 h following an increase of plasma osmolality, increased salt appetite is a delayed behavioral change which may take days to emerge. Furthermore, hyponatremia *per se* is not the prime trigger for increased salt intake [38]. In fact, rather than osmotic stimuli, hypovolemia (either true or effective), which is detected by peripheral baroreceptors [39, 40] and augmented levels of aldosterone and angiotensin II [41], are more likely to play leading roles in promoting enhanced salt appetite. Putative or documented brain structures implicated in salt appetite are numerous and their full description would stretch beyond the framework of this review. Extensive reviews on the topic can be found in [42, 43].

### Control of natriuresis

Handling of sodium excretion by the kidney is complex, involves a great number of humoral and neurohumoral mediators dedicated at defending the effective arterial blood volume. More specifically, in patients who display “effective hypovolemia”, either caused by systemic arterial vasodilation or a decrease in cardiac output, arterial underfilling represents the unifying determinant which triggers this cascade of neurohormonal responses. Alongside the secretion of AVP, the plasma renin-angiotensinogen-aldosterone system (RAAS) is activated. Renal arterial vasoconstriction ensues causing a fall in the glomerular filtration rate. This phenomenon, combined with the direct effect of enhanced angiotensin II activity, promotes proximal tubular sodium reabsorption. In addition, arterial underfilling also unleashes the activation of the sympathetic nervous system (SNS) which acts as yet another major player. Not only is the SNS connected with non-osmotic vasopressin stimulation, but is also closely intertwined with the RAAS. The SNS interacts with the latter by stimulating the release of renin. Activation of the SNS also results in marked renal vasoconstriction through α-adrenoreceptor stimulation, further increasing renal arterial resistance while decreasing glomerular filtration rate and renal blood flow [44]. This complex interplay between AVP, the SNS and the RAAS in the setting of arterial underfilling drives the emergence of an edematous state. Furthermore, it serves as physiopathological basis which reconciles the coexistence of increased ECFV and arterial underfilling or “effective hypovolemia” [45–47].

### Plasma and urine osmolality: bedside calculation and laboratory assessment

Central to the interpretation of hyponatremia is the comparison of plasma and urine osmolality. Both plasma and urine osmolality may be measured by determining their freezing point. Given that the freezing point of a solution compared to distilled water is linearly related to its osmotic pressure, its osmolality is hence readily extrapolated. Osmometers are run through this principle and provide accurate assessment of plasma and urine osmolality [48]. Since serum osmolality measurement is seldom performed on a daily basis, multiple models for predicting plasma osmolality based on routine biochemical assessment have been proposed. The following equation has emerged as both the most widely used bedside calculation method and a robust determination of plasma osmolality [49]:

$$ \begin{aligned} \text{POsm}_{c} & = \, 2 \, \times \, \text{PNa} \, + \, \text{Plasma urea} + \, \text{Glycemia (units expressed in mmol/L)} \\ \text{POsm}_{c} & = \, 2 \, \times \, \text{PNa} \, + \, \text{Blood urea nitrogen/2.8} + \, \text{Glycemia/18 (PNa expressed in mmol/L, blood urea nitrogen and glycemia expressed in mg/dL)}. \end{aligned} $$

Boasting simplicity as one of its mains assets, this equation nonetheless carries several pitfalls. First, plasma urea is not an effective osmolyte in all other instances than severe renal failure. However its incorporation enhances the precision of the equation, when compared to plasma osmolality [49]. Second, whenever additional effective osmoles (whether endogenous or exogenous) are present, the calculated plasma osmolality is bound to be inferior to the measured osmolality, thus generating an osmolal gap (see below for details). Expressed otherwise, when a measured osmolality yields a low value, one is ascertained that the patient’s patient plasma is hypoosmotic. In contrast, when faced with a low calculated plasma osmolality, a physician should rule out the possibility of an additional solute (a rare instance in clinical practice) before confidently reaching the same conclusion. In all other cases the formula produces results which fall within 5–10 mOsm of the measured osmolality [28]. Likewise, urinary osmolality may easily be inferred from simple biochemical assessment through the following equation [23]:

$$ \begin{aligned} \text{UOsm}_{c} & = \, 2 \, \times \, \left( {\text{UNa} \, + \text{UK}} \right) \, + \, \text{Urine urea} + \, \text{Urine glucose (where UNa stands for urinary sodium, UK for urine potassium, units expressed in mmol/L)} \\ \text{UOsm}_{c} & = \, 2 \, \times \, \left( {\text{UNa} \, + \text{UK}} \right) \, + \, \text{Urine urea nitrogen/2.8} + \, \text{Urine glucose/18 (units expressed in mg/dL)}.\end{aligned} $$

Similar to plasma osmolality calculation, solutes that have restricted permeability across the membrane of the distal nephron and collecting duct are accounted for in the calculation. With respect to urea, this assumption is only partly true. For reasons beyond the scope of this review, urea is indeed an effective osmole in the distal tubule. In the collecting duct, urea only fulfills this criteria in two conditions, namely in the presence of AVP and when the rate of urine urea excretion is high [23].

## Regulation of brain volume in response to hyponatremia

### Hyponatremic encephalopathy

Since the brain is confined in an inextensible, rigid structure, cell volume regulation is crucial as any excessive increase in cell volume resulting in brain swelling over a threshold of 10 % equates with severely elevated intracranial pressure, placing the patient at risk of serious brain injury and, ultimately, brain herniation [50, 51]. Furthermore, the intracranial pressure–volume exhibits an exponential relationship so that even a minimal increase of the intracranial volume translates into a dangerously augmented intracranial pressure, once this threshold has been reached [52]. This tragic chain of events occurs if adaptive responses are overwhelmed and the “regulatory volume decrease” (as detailed below) mechanism fails to quell the increase in brain cell volume, such as in the setting of profound and abrupt decrease of PNa levels. Brain edema causes the reduction of cerebral blood flow, which in turn results in cerebral ischemia, along with diminished cerebral spinal fluid production [53]. Accordingly, MRI imaging exhibits patterns suggestive of cytotoxic edema both in clinical and experimental conditions [54, 55].

### Focus on the relevant physiology: brain compensation mechanisms to hyponatremia

Conversely, if the fall in PNa levels is gradual, such as in the setting of chronic hyponatremia, the brain cells are able to elicit counteracting mechanisms in order to fend off any excessive water uptake. The most immediate response consists in the shifting of liquid from the interstitial space to the cerebral spinal fluid, a mechanism generated by the difference in hydrostatic pressure between the two compartments [56]. When glial cells are subjected to hypoosmotic stress, a swelling of these cells is initially observed. In fact, the influx of water across the blood–brain barrier inside the astrocytes is likely to be facilitated by aquaporin water channels, namely AQP1 and AQP4 [57, 58]. The pattern of AQP expression is restricted to astrocytes and ependymal cells, and they have been located predominantly in the foot processes of the former, at the interface of the brain and the major liquid compartments [59]. Consequently, water is routed selectively inside glial cells, thus sparing neurons from edema. Accordingly, AQP4-null animal models are protected against brain edema when challenged with hypoosmotic hyponatremia [60–62]. When placed in a hypoosmotic milieu, astrocytes are able to activate a mechanism known under the term of “regulatory volume decrease” which impedes the influx of water, regardless if the osmotic gradient is unfavorable. The first step of this adaptive process requires the extrusion of inorganic ions by glial cells, primarily potassium (K+) and chloride (Cl−) and represents an energy dependant process powered by the Na+–K+-ATPase system [63]. However, this counter-regulatory mechanism wanes within a few hours with brain cells even gradually recovering their initial electrolyte content. In a second phase, the compensatory process involves mainly the extrusion of organic osmolytes which drives an obligatory efflux of water [64, 65]. In experimental studies, the loss of electrolytes accounts for more than 90 % of the osmotically active agents responsible for brain volume regulation over the first 24–48 h, thereafter the efflux process relies on organic osmolytes, in case hyponatremia is sustained [66]. The time lapse required for the brain to expel osmotic moieties also serves as a basis for discriminating between acute (<48 h) and chronic (>48 h) hyponatremia. The key role and nature of organic osmolytes involved have been highlighted in multiple in vivo studies. They include myoinositol, taurine, glycerophosphorylcholine and glycerophosphoryl- ethanolamine, creatine and phosphocreatine with various regional and temporal patterns depending on the experimental model and the type of organic osmolyte [50, 54]. Their delayed time course, in contrast to electrolytes, relates to the fact that transcription and translation of transporters and upregulation of synthesis enzymes of organic osmolytes represent an obligatory and slow process before they can intervene in the cell volume regulation process [9].

### Risk factors associated with poor tolerance to hyponatremia

Accordingly, the two primary determinants which dictate the severity of hyponatremia-related symptoms are the level of hyponatremia and the rapidity of the fall of PNa levels. Life-threatening neurological symptoms are seldom encountered when PNa levels are maintained over 125 mmol/L. As outlined above, tolerance is expected to be poor whenever the decline in PNa levels outpaces the compensatory mechanisms. As a consequence, although there is no universal consensus regarding its definition, a PNa level inferior to 125 mmol/L, has recently been recognized as the biochemical threshold fulfilling the criteria for profound hyponatremia by two independent panels of experts [67, 68]. Next to these pivotal factors, other risk factors may aggravate the outcome of hyponatremia. For instance, young female patients are more inclined to develop severe neurological symptoms and seizures related to hyponatremia have been reported with PNa thresholds as high as 130 mmol/L. However, these cases mainly emanate from a single investigating team [69, 70] and the mechanisms responsible for this female preponderance have yet to be fully elucidated, although experimental studies have highlighted the synergistic and deleterious effects of vasopressin and estrogen on Na–K-ATPase activity in the CNS, a critical component of the adaptive response to hyponatremia of the brain [71, 72]. The co-occurrence of hypoxia along with severe hyponatremia also portends a poor prognosis and a possibly enhanced risk of permanent brain damage [69]. In experimental settings, animals manifest increased cerebral edema and reduced cerebral perfusion as a result of the combined effects of hyponatremia and hypoxia. Furthermore, hypoxia has been shown to suppress the compensatory mechanisms elicited by hyponatremia [73]. More specifically, the Na–K-ATPase activity is virtually obliterated when a hypoxic insult is superimposed upon hyponatremia [74].

### Osmotic demyelination

#### Overcorrection of hyponatremia and the risk of osmotic demyelination

By reviewing 12 cases of autopsy-proven cases of OD, Norenberg et al. were the first to hypothesize a connection between the overcorrection of PNa and the occurrence of OD [75]. In their landmark study, the PNa increased by at least 20 mmol/L over the course of 1–3 days prior to the emergence of the symptoms related to OD in all 12 patients. Experimental data [76, 77] and other clinical series [78] soon confirmed this theory. Sterns, reporting on 8 patients with OD, showed that the PNa correction rate had exceeded 12 mmol/L/day in all cases [78]. Focusing on patients with severe hyponatremia, further investigation from the same team yielded no case of OD, provided the PNa sustained correction rates lower than 12 mmol/24-h and 18-mmol/L/48-h [79]. Of note, patients presenting with profound (<120 mmol/L) and chronic (>48 h) hyponatremia also carry a greater potential for OD [70, 80]. However, since these results have been issued, there has been a steady stream of reports documenting cases of OD although the PNa correction rate was kept within these marks [81–86]. One important implication of these reports is the careful scrutiny of other risk factors for OD.

#### Pathophysiology of osmotic demyelination

The most prominent neuropathological finding in OD consists in the symmetrical destruction of myelin shafts and oligodendrocytes, perhaps through apoptosis, typically in the basis pontis [87, 88]. The inflammatory process is at best limited to scatters of macrophages in the demyelinated area while neurons are usually spared until the advanced stages of the disease. The term central pontine myelinolysis (CPM) refers to the preferential location of the disease and was devised after the description of the index case [89]. Why the pons is a privileged target for OD is certainly the result of its neuroanatomy: an intricate network of gray and white fibers. As gray fibers are well endowed with capillaries, the adjacent white matter is thus vulnerable to circulating neurotoxic factors, in this case the osmotic gradient generated by a hasty rise in PNa [90]. The term is in fact misleading as areas from the CNS outside the pons are susceptible to OD. Extra-pontine myelinolysis, which usually involves the sub-cortical gray matter may thus develop either concurrently with CPM (31 %) or may represent the exclusive manifestation of OD (22 %) [91]. Next to the pons, the most frequently described locations of OD involve the cerebellum, the lateral geniculate bodies, the external capsule, the hippocampus, the putamen, the striatum and the thalami [92]. Many aspects of the physiopathology of OD are unsettled. It is believed that an overshoot in the correction of PNa is followed by a swift reaccumulation of electrolytes (K+ and Cl−) which had initially been extruded by brain cells in a bid to offset brain edema in the early stages of hyponatremia [64]. At variance, a protracted lapse of time is mandated for these cells to regain their original levels of osmolytes [66, 93]. There is a good level of evidence suggesting that the disequilibrium between the levels of osmolytes and electrolytes is closely connected to OD, but the precise sequence of events linking this imbalance and the occurrence of demyelination is still under scrutiny. The breakdown of the blood–brain barrier is another characteristic feature of OD and restoring its integrity using glucocorticoid therapy is known to mitigate OD in the experimental setting [94, 95]. How OD may participate to the disruption of the blood–brain barrier and, reciprocally, how the latter may contribute to demyelination remain areas of speculation. Finally, the microglia, which infiltrates the areas of demyelination, has been entertained as yet another detrimental actor through the inflammation it generates [96, 97]. Experimentally, reversal of its activation confers protection against demyelination [98, 99].

#### Other risk factors for osmotic demyelination

Strikingly, long before OD was recognized as a complication of the overcorrection of hyponatremia, investigators had noted that alcoholic patients were especially prone to this complication. In fact, three out of four patients from the original description of the disease were alcoholics [89] and other series have reported rates of alcoholism reaching 70 % [91]. Patients may even develop OD despite an optimal correction of hyponatremia or in the absence of preexisting plasma sodium disorders. In this case they usually disclose a history of alcohol abuse [100–102]. The mechanism by which alcohol consumption favors the occurrence of OD is hitherto unknown. Likewise, malnutrition is also very prevalent among patients with OD [92].

Liver disease and liver transplantation represent potent risk factors for OD [103]. In that latter condition, up to 3.5 % of patients have been reported to be affected by OD [104]. Furthermore, liver transplantation ranks third in term of comorbid condition associated with OD [105]. In this setting, OD is expected to occur early in the course of the patient’s post-operative management, usually within a week [106]. Liver transplantation is thought to give rise to an abrupt correction of preexisting hyponatremia thus paving the way for OD [104]. Calcineurin inhibitors, a cornerstone of patient’s immunosuppressive regimen in transplantation, possess neurotoxic effects on white matter, and may further potentiate the risk of OD [104].

Hypokalemia also predisposes to OD [107]. In reality it is potassium repletion, in case of coexisting hypokalemia and hyponatremia, which carries the risk of OD [108]. Indeed, since PNa levels are determined by total exchangeable sodium and total body water but also by total exchangeable potassium [10], any potassium delivery to the patient will result in an increase in PNa levels, hence the risk of inadvertent PNa overcorrection if this phenomenon is not accounted for.

Conversely, uremia is believed to be at least partially protective against OD, probably because it enables the brain to reclaim organic osmolytes more swiftly [109]. In the experimental stage, uremic animals exhibit expeditious recovery of myoinositol and other organic osmolytes, perhaps because in response to uremia brain cells exhibit enhanced organic osmolytes transport [109]. However the validity of this concept should be mitigated by the existence of several cases of OD in the setting of uremic patients undergoing hemodialysis [110, 111].

## Clinical presentation of hyponatremia

### Symptoms associated with hyponatremia

Clinical manifestations ascribable to hyponatremia are predominantly the expression of CNS dysfunction (see Table 1). The pattern of symptoms correlates with the level of hyponatremia and whether the disorder has developed rapidly or not [112, 113]. A panel of experts has recently proposed a biochemical grading of severity of hyponatremia [68]. It forms the basis for the classification exposed herein below. In case of mild hyponatremia (PNa comprised between 130 and 135 mmol/L), symptoms may be restricted to fatigue, mild cognitive and gait impairment [114]. Caution should be exerted before dismissing as asymptomatic patients with mild hyponatremia since a meticulous examination will in fact almost always unmask subtle neurocognitive deficiencies. Reciprocally, the existence of severe neurological symptoms in contrast with only mildly reduced levels of PNa should prompt physicians to consider other cause(s) of neurological impairment. In moderate hyponatremia (PNa between 125 and 130 mmol/L), the clinical picture is dominated by non specific symptoms including headaches, nausea, emesis and abdominal cramps. When confronted with profound hyponatremia (PNa <125 mmol/L), restlessness, lethargy, confusion, delirium but also coma, seizure and brain herniation with permanent brain damage and death are to be feared [112]. Furthermore, the course of the symptoms is largely unpredictable, and the patient’s clinical condition may swiftly deteriorate in the face of modest PNa decline. Accordingly, seizures are acknowledged to be the inaugural manifestation of hyponatremia [115] and they rank as one of the foremost cause of new-onset epilepsy on the ICU [116]. Extra-neurological consequences of hyponatremia comprise hypercapnic neurogenic respiratory failure which has been described primarily in young women in the post-operative setting [117].

**Table 1:** Clinical signs of hyponatremic encephalopathy and osmotic demyelination

Hyponatremic encephalopathy^a^	Central pontine myelinolysis	Extra-pontine myelinolysis
Mild hyponatremia	Suggestive features	Movement disorders
Mild neurocognitive impairment	Quadriparesis/plegia	Extra-pyramidal syndrome
Gait impairment	Ataxia	Dystonia
Moderate hyponatremia	Nystagmus	Choreoathetosis
Headaches	Dysarthria	Myoclonus
Nausea	Ophthalmoplegia	Opsoclonus
Emesis	Dysphagia	Akinetic-rigid state
Abdominal cramps	Pseudobulbar palsy	Akinetic mutism
Severe hyponatremia	“Locked-in” syndrome	Other symptoms
Restlessness	Associated neurological features	Emotional lability
Lethargy	Impaired consciousness	Depression
Confusion	Wernicke’s encephalopathy	Paranoia
Coma		Disinhibition
Seizure		

^a^Neurological manifestations according to the severity of hyponatremia which is itself correlated to the magnitude and the rapidity of the fall in PNa

### Osmotic demyelination: clinical course

The clinical course is generally biphasic with inaugural symptoms of hyponatremic encephalopathy, as previously described, followed by a lucent interval of 1–7 days before clinical evidence of OD become manifest, but the onset has been reported to be delayed by as much as 2 weeks. Signs distinctive of CPM include ataxia, nystagmus, dysarthria, ophthalmoplegia, dysphagia, pseudobulbar palsy, flaccid followed by spastic quadriparesis [118]. When it is full blown, CPM is characterized by the “locked-in” syndrome.

Depending on the brain region compromised by OD, various clinical patterns have been depicted (see Table 1).

Extra-pyramidal symptoms including dystonia, extra-pyramidal rigidity and tremor have been the focus of reports as well as other movement disorders including choreoathetosis, myoclonic jerks and catatonia [119]. Cases of OD masquerading as parkinsonism have been recorded [119, 120].

Neuropsychological symptoms are frequently found and in as many as one patient out of four such symptoms may be the exclusive manifestation of OD [105]. Impaired consciousness and seizures are other salient features at presentation [121].

Of note, symptoms related to OD may be intertwined with other neurological syndromes complicating alcohol abuse, given the high prevalence of this condition in patients affected with OD, such as Wernicke’s encephalopathy or Marchiafava–Bignami disease [91, 92].

In the setting of OD, MRI imaging will characteristically exhibit a lesion with hyperintense changes on T2-weighted sequences accompanied by hypointense changes on T1-weighted sequences. Contrast enhancement is most frequently absent. Importantly, a normal MRI imaging does not rule out the diagnosis of OD as the aforementioned findings lag behind the clinical onset of the disease [118, 122, 123].

The prognosis in terms of survival may not be as dismal as previously reported. In a retrospective study on patients admitted in the ICU for OD, Louis et al. found a 1 year mortality rate of 31 %, markedly below the mortality rates yielded by previous reports which approximated 50 % [91, 124, 125]. Furthermore, 50 % of the surviving patients in this study had either recovered or sustained only mild forms of neurological disability, as assessed by the Rankin scale [124]. No clinical or imaging feature has been conclusively established as predicting the prognosis, although impaired consciousness upon initial presentation is usually associated with a poor outcome [121, 125]. Taken together, this data suggest that OD should not per se prompt intensivists to forego life-support measures.

### Impact of hyponatremia on the outcome of ICU patients

There exist a fair number of converging studies indicating that hyponatremia is associated with a pejorative outcome among patients in the ICU (see Table 2 for details). Hyponatremia is an independent risk factor for shortened in-hospital survival [4–6, 126, 127] and a prolonged stay in the ICU [6]. Strikingly, it has been repeatedly demonstrated that the mortality risk is correlated to the magnitude of hyponatremia [4, 6, 126, 128] and that even mildly diminished levels of PNa portend a poor prognosis [126]. As for hospitalized patients in general, a greater proportion is destined to require ICU admission or mechanical ventilation during their management in case of documented hyponatremia upon admission [129]. This is not clear whether hyponatremia is merely a reflection of the severity of the underlying illness and associated comorbid conditions or if it directly influences the course of patients [51, 130].

**Table 2:** Epidemiology of hyponatremia in the ICU and impact on outcome

First author, year of publication	Study methodology	Definition of hyponatremia	Number/total population (%)^c^	Patient characteristics associated with hyponatremia		Outcomes significantly associated with hyponatremia	
				Univariate analysis	Multivariate analysis	Studied variable	Outcome^e^
Vandergheinst, 2013	Prospective, 1 day multicentric 1265 ICUs	<135 mmol/L	1713/13,796 (12.9 %)	SAPS II SOFA ICU LOS (129 < PNa < 135) Cirrhosis (PNa < 130) Infection (125 < PNa < 135) Medical (PNa < 125)	–	Hyponatremia, according to severity and type	Increased hospital mortality (whole population)^f^
		1. Severity of hyponatremia^a^					
		2. Type of hyponatremia					
		On admission					
		ICU acquired^b^					
		3. Whole population					
Sakr, 2013	Retrospective Monocentric 1 ICU	<135 mmol/L	1215/10,923^d^ (11.2 %)	SAPSII ICU LOS Diabetes Cancer Hematological cancer AKI	–	Hyponatremia on admission	Increased hospital mortality
		On admission					
			1483/10,923^d^ (13.6 %)				
		ICU acquired^b^					
Darmon, 2013	Retrospective 13 ICUs	<135 mmol/L Severity of hyponatremia^a^ on admission	3047/11,125 (9.7 %)			Hyponatremia according to severity	Increased day-30 mortality (for moderate and severe hyponatremia)^h^

Study, year of publication	Study methodology	Definition of hyponatremia	Number/total population (%)^c^	Patient characteristics associated with hyponatremia		Hyponatremia as a risk factor for ICU	
				Univariate analysis	Multivariate analysis	Studied variable	Outcome^e^
Funk, 2010	Retrospective multicentric 77 ICUs	= or <135 mmol/L on admission	26,782/151,486 (17.7 %)	–	–	Hyponatremia	Increased hospital mortality
Stelfox, 2008	Retrospective multicentric 3 ICUs	<133 mmol/L ICU acquired^b^	917/8142 (11 %)	–	Age Setting	Hyponatremia	Increased ICU mortality
					Neurosurgical		
					Surgical		
					Trauma patients		
					APACHE II GCS score Glucose level K+ >5 mmol/L T° > 37.3 °C and T° °C < 35 °C		
Bennani, 2003	Retrospective monocentric 1 ICU	<130 mmol/L Severity of hyponatremia^g^ on admission	300/2188 (13.7 %)			Hyponatremia according to severity	Increased hospital mortality (PNa < 125 mmol/L)
deVita, 1990	Retrospective monocentric 1 ICU	<135 mmol/L on admission	24/98 (24.5 %)	_	_	–	–

*ICU* intensive care unit

^a^As defined by mild hyponatremia: PNa comprised between 130 and 134 mmol/L, moderate hyponatremia: PNa comprised between 125 and 129 mmol/L, severe hyponatremia: PNa <125 mmol/L

^b^Acquired during the ICU stay

^c^The variables are number of patients with hyponatremia, total number of patients admitted during the study period, percentage of patients with hyponatremia (%)

^d^Number of patients with hyponatremia, total number of patients admitted during the study period, percentage of patients with hyponatremia (%) upon ICU admission and acquired during the ICU stay

^e^In each case there is a positive correlation between the studied variable and the defined outcome

^f^Patients with hyponatremia admitted in the ICU during the study day or prior to the study day

^g^As defined by mild hyponatremia: PNa comprised between 125 and 129 mmol/L, moderate hyponatremia: PNa comprised between 120 and 124 mmol/L, severe hyponatremia: PNa <120 mmol/L

^h^As defined by moderate hyponatremia: PNa comprised between 125 and 129 mmol/L, severe hyponatremia: PNa <125 mmol/L

Hyponatremia also exerts a negative impact on patient morbidity and mortality across various specific conditions frequently encountered in the setting of the ICU. Patients admitted for community-acquired pneumonia often exhibit an AVP-dependent impairment of water excretion [131]. Furthermore, they fare worse whenever hyponatremia is present upon admission [132].

In the context of heart failure, the co-occurrence of hyponatremia is consistently associated with protracted hospital stay [133] and predicts reduced short-term and long-term survival [134, 135]. Hyponatremia is also intimately connected to advanced liver disease and a heightened mortality risk. Clinical studies have repeatedly pointed to hyponatremia as one of the culprits in the pathogenesis of hepatic encephalopathy, possibly by thwarting the astrocytes compensatory mechanisms, including a depletion in brain osmolytes [136] and by generating a state of low-grade cerebral oedema [137]. Recently, in large retrospective study in patients with chronic kidney disease, hyponatremia was found to be independently associated with increased mortality, irrespective of concomitant heart failure, and the survival rates of patients paralleled that of the sodium plasma levels [138].

In the field of neurological disorders, hyponatremia should not be dismissed as a benign finding, as even small decreases of PNa have been shown to be associated with a compromised prognosis. As a matter of fact, hyponatremia has been proven an independent predictor of mortality in various settings such as patients with stroke [139], adults with tuberculous meningitis [140] and children affected with pneumococcal meningitis [141]. As for patients with sub-arachnoid hemorrhage, the risk of vasospasm is increased whenever hyponatremia coexists [142]. This is not surprising given the exquisite sensitivity of the brain to elevated intracranial pressure, the latter being (negatively?) impacted by hyponatremia.

## Etiologic diagnosis

### Etiologic diagnosis strategy and conditions associated with hyponatremia

**Fig. 1:**
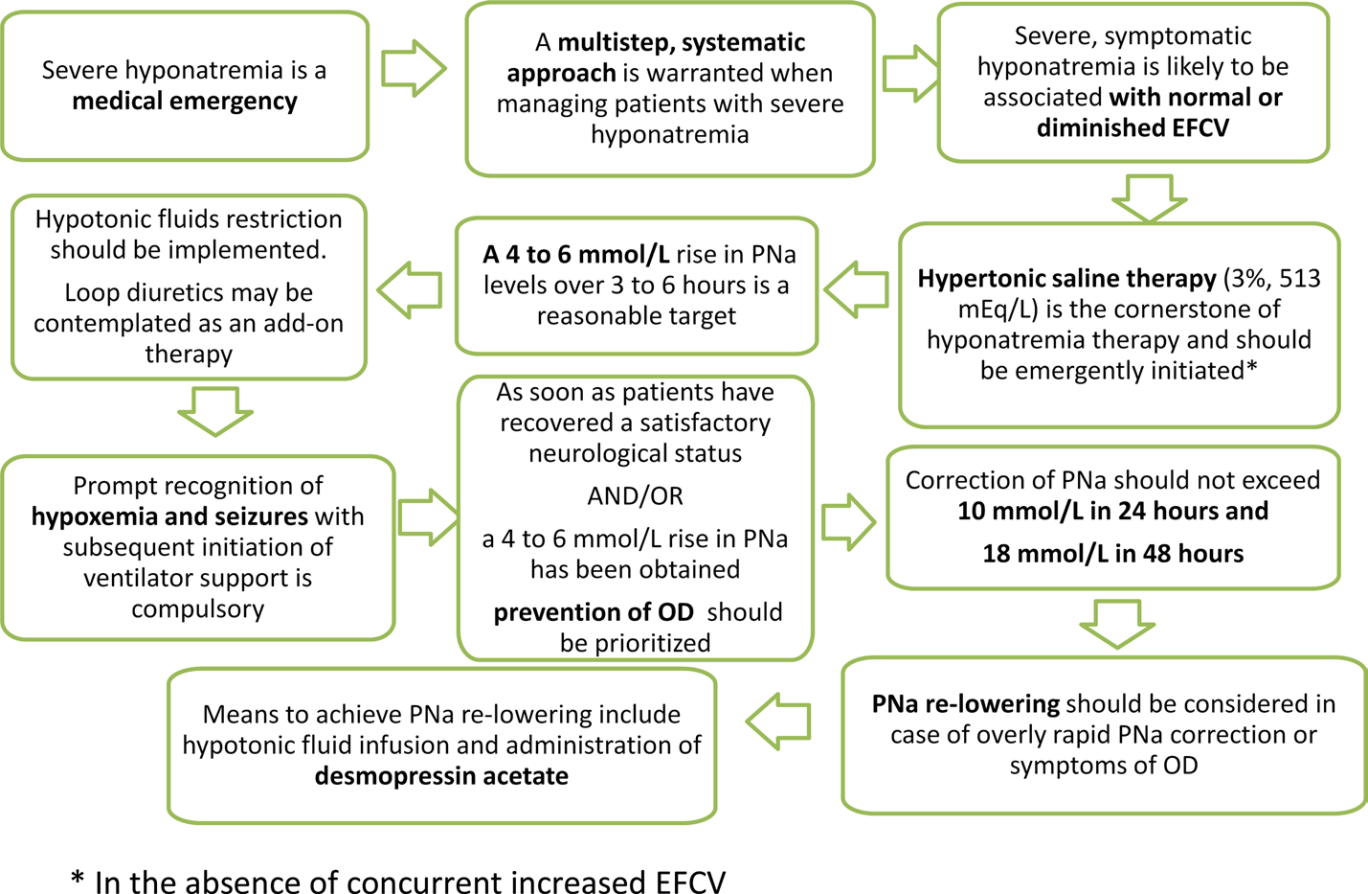
Key points when managing severe hyponatremia in the ICU. *Asterisk* in the absence of concurrent increased EFCV.* a* A rare instance in routine clinical practice.* b * High water intake along with increased AVP levels may coexist. CKD chronic kidney disease, SIADH syndrome of inappropriate antidiuretic secretion, ${\rm NA}_{\rm u}^{+}$urine sodium concentration

**Fig. 2:**
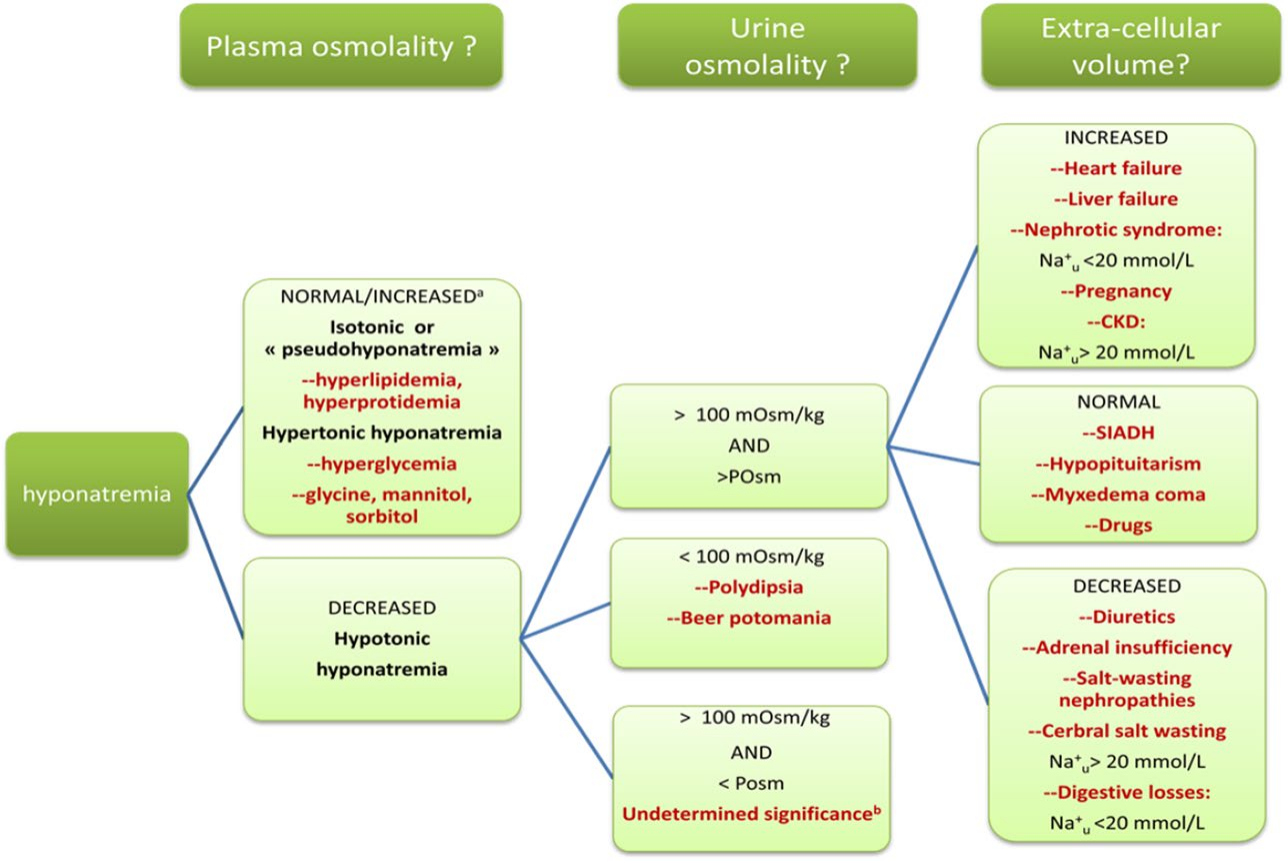
Etiological diagnostic strategy. *CKD* chronic kidney disease, *SIADH* syndrome of inappropriate antidiuretic secretion, ${\text{Na}}_{u}^{ + }$ urine sodium concentration. *a* A rare instance in routine clinical practice. *b* High water intake along with increased AVP levels may coexist. *Asterisk* in the absence of concurrent increased EFCV

Unraveling the cause(s) of hyponatremia requires a sequential approach as depicted in Figs. 1 and 2. The algorithm depicted in Fig. 2 is based on published works and guidelines [143, 144] and studies have confirmed its appropriate diagnostic accuracy [145] provided that a meticulous step by step analysis is performed and that physicians are aware of some of its pitfalls [145]. However, physicians should not be deterred by the apparent complexity of the algorithm. As highlighted in the following sections, most of the issues derived from it can be solved at the patient’s bedside, the great bulk of patients belong to the subgroup exhibiting hypotonic hyponatremia and those with ill-tolerated hyponatremia either retain a normal ECFV or display a diminished ECFV.

### Hyponatremia in the setting of normal or increased tonicity

As Na is the preponderant solute in the extracellular fluid, PNa also serves as the prime determinant of serum tonicity. It follows that hyponatremia almost universally indicates a state of hypotonicity. In other words, the vast majority of cases of hyponatremia (especially when profound) are hypoosmotic hyponatremia, a term largely preferable to the so-called “true hyponatremia”. However, two exceptions to this rule may be delineated.

#### Spurious isotonic hyponatremia or so-called “pseudohyponatremia”

Cell hydration and volume is not affected in this condition. Spurious isotonic hyponatremia represents a phenomenon which arises in case of a major increase of the solid fraction of the plasma stemming from either a major increase of lipids or proteins. As a result, the plasma water fraction is contracted and the PNa level may deceivingly appear to be lowered. Hence the confusing term “pseudohyponatremia” which is meant to allude to the fact that the intracellular volume is sustained despite lowered PNa values. As a result, neurological symptoms should not be interpreted as a manifestation of the diminished levels of PNa. They are in fact more likely be related to hyperviscosity. The phenomenon of isotonic hyponatremia (“pseudohyponatremia”) can be suspected very easily from the outset simply by concurrently measuring the levels of protein levels and by paying attention to the serum’s aspect: in the background of hypertriglyceridemia, it will almost certainly appear to be opalescent. Indeed, noticeable isotonic hyponatremia is solely observed when protein or lipid concentrations are extraordinarily high. Alternatively, the genuine levels of PNa can be deduced by simple computations. In fact, this pitfall is uncovered in most hospitals because PNa levels are assessed by using direct ion-selective electrode for measuring PNa levels instead of indirect ion-selective electrode. The latter method, resorts to a dilution phase, as assumes a constant non-water fraction of 7 %. It follows that indirect ion-selective electrode measurements will provide a factitious decrease in PNa levels when the solid component of serum is augmented, at variance with direct ion-selective electrode testing which do not require pre-analytic dilution and takes into account the variable volume share in lipids and colloidal proteins with respect to total plasma volume. [146, 147].

#### Hypertonic hyponatremia

The intracellular compartment is dehydrated and its volume decreased in this condition. Hyponatremia with increased osmolality is to be considered when compounds other than sodium, whether endogenous or exogenous, create an osmotic drive causing the relocation of water from the intracellular compartment to the extracellular compartment.

Hyperglycemia, such as found in ketoacidosis and the hyperglycemic hyperosmolar state is the best known cause of hyponatremia in relation with endogenous compounds. When there is a severe degree of hyperglycemia, in the absence of insulin, this causes a rise in the plasma osmolarity thus forcing water to shift from the intracellular compartment to the extracellular compartment [148, 149]. This relocation of the water content explains the observed fall of PNa, a situation typical of hyperosmotic hyponatremia.

Of note, the works of Katz et al. have popularized the use of a correction formula which predicts a 1.6 mmol/L reduction in PNa levels whenever the glucose levels increases by 5.6 mmol/L [150]. This computation should nonetheless be handled with care as it may tend to depreciate the true drop in PNa and another corrective computation has been devised [23, 151]. In order to circumvent this pitfall, plasma osmolarity should be the preferred monitoring parameter during the patient’s management, as PNa may appear to increase while in fact plasma osmolarity is undergoing an expeditious decline, as glucose levels fall.

#### The post- transurethral resection of the prostate (TURP) syndrome

Examples of exogenous “effective osmoles” include glycine, mannitol and sorbitol. The “post-TURP syndrome” represents yet another well recognized condition where the presence exogenous osmoles (glycine, mannitol and sorbitol) give rise to hyponatremia. Indeed, during post-transurethral resection of the prostate (TURP), it is custom to instillate a solution of hypotonic fluids containing any of the aforementioned solutes though a Foley catheter for electrocoagulation purposes. These irrigation solutions were designed to replace distilled water, the reabsorption of which caused massive hemolysis and acute kidney injury [152]. However, in case the posterior wall of the prostate is breached or a venous plexus is severed during surgical manipulations, the ensuing intraperitoneal extravasation of the irrigation fluid, or its direct reabsorption in the circulation, may cause acute hyponatremia in the face of an osmolality which varies over time from hyper to hypoosmolality [153]. Indeed two sequential mechanisms concur to determine hyponatremia: (1) glycine and sorbitol are initially restricted in the extracellular compartment, at least in the very early stages following the administration of the lavage fluids. Both particles are osmotically active and thus prompt water to shift from the intracellular compartment into the extracellular compartment thus causing hyponatremia. (2) Glycine and sorbitol penetrate into cells within two hour following the administration of lavage fluids where they are metabolized. (3) The net balance at this time point is a sheer gain of water along with hypoosmotic hyponatremia. Brain swelling may now ensue, potentially jeopardizing the patient’s neurological status [23, 154]. By contrast, mannitol does not undergo cell metabolization and promotes hyponatremia through direct osmotic or “translocational” effect with no concurrent major change in plasma osmolality [155, 156]. Although the setting is usually sufficiently suggestive to prompt the diagnosis, the osmolar gap may be a helpful adjuvant in unmasking the presence and indirectly determining the concentration of the solute used. If the irrigation solution contains glycine, severe encephalopathy is to be feared, as glycine metabolism yields ammonemia [157]. Since its first description in the post-operative course of TURP, similar complications have been described following in a wide range of endoscopic surgeries [156].

In “conclusion”, although the multiplicity of etiologies and the somewhat intricate nature of mechanisms related to normotonic or hypertonic hyponatremia may seem bewildering at first, physicians may salvage consolation in the fact that these conditions are rare, cause only mild to moderate hyponatremia and can be readily ruled out by a simple and expeditious clinical and biological assessment. Once the diagnosis of hypotonic hyponatremia is ascertained, several steps must be undertaken in order to elucidate the precise mechanism of this disorder.

### Hyponatremia and hypotonic urine: look out for hypotonic fluid intake

The next phase is to evaluate urine tonicity on order to discriminate patients with impaired urine diluting mechanisms, a vast majority of patients in the ICU, from the small subset of patients who retain diluting abilities [3]. In the event of hyponatremia, renal urinary diluting capacities are usually deemed satisfactory whenever the urinary osmolality is inferior to that of plasma and, at best, if urine osmolality does not exceed 100 mOsm/kg [145].

Hypotonic hyponatremia in association with hypotonic urine is strongly suggestive of an excessive water intake, including psychogenic polydipsia, a psychiatric disorder which may affect up to 25 % of patients diagnosed with schizophrenia [158]. Given that in physiological conditions, and provided that patients are on a normal diet, the kidneys boast a maximum excretory capacity of 10–15 L/day it is unlikely that polydipsia *per se* is sufficient to induce hyponatremia, even when vast amounts of hypotonic fluids are ingested by the patient. In fact, clinical investigations have pointed out subtle defects in the maximum urinary dilution and free water clearance in patients with polydipsia and hyponatremia, perhaps in relationship with an enhanced sensitivity to arginine vasopressin [147, 148]. What’s more, polydipsic patients exhibit increased thirst across a wide range of osmolality, compared to controls [148]. The underlying mechanisms responsible for these alterations in water homeostasis remain to be unfolded, yet hyponatremic polydipsic schizophrenic patient display structural changes and neuroendocrine impairment affecting the anterior hippocampus which may serve as an anatomical substract for future investigations [149, 150]. Finally, an additional level of complexity arises from the comorbidities and therapies frequently associated with schizophrenia which may be involved in the pathogenesis of hyponatremia, first and foremost being the prescription of antipsychotic and antidepressant [151].

In a similar vein, “beer potomania” may also result in hyponatremia together with lowered urine osmolality. In this condition, patients typically have a history of low solute intake and a diet deprived of protein whereas they ingest vast amounts of beer, a largely hypotonic beverage. Consequently, these patients are unable to produce the osmolar load which drives the free-water excretion in physiological conditions [159]. In the face of large volumes of hypotonic fluid intake, this equates with hyponatremia. A situation that can be very much likened to beer potomania is the “tea and toast” syndrome. Similar to potomania, patients –almost universally elderly, incapacitated subjects- are fed on low protein diet (“toasts”), only in this case the hypotonic beverage consists in tea.

### Hyponatremia with impaired urinary dilution: assess the patient’s extracellular fluid volume status

Once the previous steps have been cleared, the most plausible case-scenario left is hypoosmotic hyponatremia along with non-maximally diluted urine. In these circumstances, a defect in tubular diluting functions, caused by the secretion of AVP, is likely to underpin the occurrence of hyponatremia and urine osmolality is typically expected to be superior to plasma osmolality. Notable exceptions to this interpretation include conditions which interfere with normal tubular function with the most prominent examples being the use of thiazide diuretics and severe kidney disease. Finally a urinary osmolality superior to 100 mOsm/kg, but inferior to that of the plasma frames an area of uncertain interpretation. In this case, both high water intakes along with low solute intake may coexist with excessive vasopressin action [68, 160].

The clinical conundrum can thereafter be restated as follows: what is the mechanism that drives the secretion of AVP? Assessment of ECFV is of paramount importance and may provide meaningful insights to that question. It should include a gross appreciation of the total body sodium stores and sources of hypotonic fluids intake, including of iatrogenic origin. Most of all it should allow for the discrimination of 3 subsets of patients with decreased, increased or normal ECFV. In patients with decreased ECFV, AVP production is directly induced by the hemodynamic stimulus resulting from “true hypovolemia”. As for patients with increased ECFV, a category largely dominated by conditions related to heart failure or cirrhosis, “effective hypovolemia” provides the grounds for increased AVP secretion. Finally, in euvolemic patients, AVP secretion is unconnected to its usual physiological triggers.

Hyponatremia with normal ECFV overlaps extensively with the syndrome of inappropriate antidiuretic (AVP) hormone secretion. In order for this diagnosis to be contemplated the following criteria should be fulfilled: hypotonic hyponatremia, urine osmolality in excess of plasma osmolality, euvolemia, and normal renal and adrenal function [161]. However distinguishing euvolemic patients from mildly hypovolemic patients based solely on clinical grounds is notoriously challenging [145, 162, 163].

Dosing AVP is of limited usefulness, as the levels of plasma of AVP have been proven to fluctuate unpredictably and are often augmented in patients with both true hypovolemia (decreased ECFV) and effective hypovolemia (increased ECFV), as a result of a non-osmotic stimulus, as well as in the syndrome of inappropriate antidiuretic hormone secretion (SIADH), which remains in essence, a diagnosis of exclusion. Nevertheless, other biological features may represent a valuable aid for physicians investigating the presence of SIADH. They include lowered plasma uric acid and blood urea nitrogen, and failure to correct PNa levels following the infusion of 0.9 % saline solution [144, 164]. Uric acid stands out as, perhaps, the most useful static biological parameter. Reduced levels of uric acid levels are thought to result from the mild volume expansion which occurs at the onset of SIADH, as well as from increased urinary excretion [165, 166]. However, the coexistence of hyponatremia and lowered uric acid levels may also be witnessed in other conditions unrelated to SIADH such as the use of diuretics, potomania and cirrhosis [166, 167].

When the physician feels unconfident whether the patient’s ECFV status is normal or decreased, a simple diagnostic test consists in the infusion of 0.9 % saline (NaCl) solution. In the event of SIADH, the infusion of the 0.9 % NaCl solution will not ameliorate and may even worsen PNa levels, and, in any case, an immediate rise in natriuresis will be observed. This phenomenon which goes by the name of “desalination” describes the process whereby following the administration of isotonic saline fluids and due to the action of AVP, the sodium load is excreted in hypertonic urine while the free water content is reabsorbed in the patient’s body. Conversely, the administration of a 0.9 % NaCl solution will tend to normalize the PNa in hypovolemic patients, with no appreciable and immediate modification of the urinary concentration in sodium, until ECFV is restored. Thereafter, a delayed rise in sodium excretion in order to balance sodium intake will be manifest [168]. Nevertheless, physicians will find consolation in the fact that patients admitted in the ICU for profound and/or severe symptomatic hyponatremia are bound to display obvious abnormalities of the ECFV status, whether increased or decreased.

#### Patients with normal ECFV: when should SIADH be suspected?

##### Pathophysiology of SIADH

In this setting, the primary physiopathological mechanism responsible for hyponatremia is the disconnection of AVP secretion from its usual osmotic and hemodynamic stimuli. AVP triggers water reabsorption via the collecting ducts, thus promoting a state of body water overexpansion which, in turn, leads to hyponatremia by dilution of the plasma PNa content. Patients suffering from SIADH are devoid of overt signs of hypervolemia because compensatory mechanisms aiming at restoring a normal extracellular fluid volume are elicited [169]. The pivotal mechanisms consist chiefly in the augmented secretion of atrial natriuretic peptide and the suppression of renin and aldosterone secretion [170]. The net effect is a transient negative sodium balance due to a transient increase in natriuresis (in order to restore a normal ECFV but at the expense of lowered tonicity).

At a later stage, a down-regulation of AVP receptors at the surface of the collecting ducts blunts the response to increased AVP levels, a phenomenon acknowledged under the term of “AVP antidiuresis escape” [171, 172]. Consequently, this intricate interplay between AVP secretion, AVP-resistance and natriuresis allows PNa to settle at a steady level and to sustain euvolemia (See Table 3). Since its description in 1957 in two patients with bronchogenic carcinoma [173], SIADH has been described as an epiphenomenon of a broad range of conditions and diseases which are recapitulated in Table 3. Finally, there is an ever-expanding list of agents that may cause hyponatremia through various SIADH-related mechanisms, namely by enhancing the release of AVP (neuroleptics, anti-depressant drugs and anti-neoplastic drugs), by potentiating (carbamazepine), or even by mimicking its action on collecting ducts (oxytocin) [174].

**Table 3:** Causes of SIADH

Paraneoplastic production of AVP	Eutopic production of AVP/ enhanced hypothalamo–pituitary release of AVP
SCLC non-SCLC	CNS disorders
Head and neck cancer	Infectious: meningitis, encephalitis, brain abscess
Mesothelioma	Vascular: SAH, subdural hematoma, thrombosis
Stomach carcinoma	Multiple sclerosis
Duodenum carcinoma	Acute intermittent porphyria
Thymoma	Guillain-Barré syndrome
Lymphoma	Shy-Drager syndrome
Olfactory neuroblastoma	Schizophrenia
Bladder carcinoma	Pulmonary disorders
Sarcoma	Pneumonia, tuberculosis
Potentiation of AVP effects	Pneumothorax, atelectasis
Carbamazepine	Asthma, cystic fibrosis
Sodium valproate	Positive pressure ventilation
Cyclophosphamide	Drugs
	Neuroleptics
	TCC, MAOI, and SSRI antidepressants
	Antineoplastic drugs
	Others
	Hypopituitarism
	Severe hypothyroidism
	Nausea, pain

*AVP* arginine vasopressin, *SCLC* small cell lung cancer, *SAH* subarachnoidal hemorrhage, *TCC* tricyclic antidepressant, *MAOI* monoamine oxydase inhibitor antidepressant, *SSRI* selective serotonin uptake inhibitor antidepressant

##### Endocrine disorders and inappropriate AVP secretion

Before claiming that a patient’s low level of PNa may be imputed to SIADH, one must keep in mind that various endocrine disorders induce hyponatremia the features of which may masquerade as SIADH, at least at first glance. Hypothyroidism has long been viewed as a potent cause of hyponatremia which is regarded as one of the prime features of myxoedema coma [175, 176]. Mechanistic features suggestive of SIADH including impaired urinary dilution, failure to excrete large volume of free water have and, in some cases, reports of elevated AVP plasma levels have reinforced this notion. The relevance of hypothyroidism-induced hyponatremia has, however, recently been challenged by clinical studies which did not find any significant association between the two clinical entities, at least in the mild form of this endocrinopathy [177]. Therefore, one should not ascribe hyponatremia to hypothyroidism before other causes of hyponatremia have been carefully scrutinized. Patients affected with isolated glucocorticoid deficiency, a hallmark of hypopituitarism, usually retain a normal volemic status, in contrast to patients with primary adrenal insufficiency. Yet, hyponatremia is a frequent finding in these cases [178] and its biochemical characteristics may prove to be indistinguishable from those of SIADH [179]. Both AVP-dependent and AVP-independent pathways have been postulated as the possible mechanism underlying hyponatremia in these cases [180, 181].

##### Inflammation as a potent stimulus for AVP release

In the post-operative setting, patients often cumulate various risk factors placing them at high risk for the development of SIADH. Nausea, pain, hemodynamic instability, neurological and pulmonary insults are believed to act as non-osmotic stimuli which enhance the release of AVP and may even prevail over AVP-inhibition generated by an hypoosmotic state [182–184]. Additionally, there has been mounting evidence that the key pro-inflammatory cytokine IL-6 may serve as the missing link between states of systemic inflammation, a hallmark of post-operative patients, and the release of AVP and subsequent hyponatremia [185, 186]. IL-6 deficient mice exhibit reduced expression of AVP [187]. Ghorbel et al. were able to demonstrate that IL-6 is not only largely co-localized in the SON and PVN but its expression is upregulated in settings of potent AVP stimulation such as dehydration [188]. Finally, by injecting a recombinant form of this cytokine to human volunteers, a team of investigators were able to establish that IL-6 exerts secretagogue action on PVN AVP secretion [189]. Pathways connecting IL-6 to vasopressin release are still under scrutiny but IL-6 has been shown to be capable of crossing the blood brain barrier. Alternatively, other cytokines may elicit the secretion of IL-6 by blood brain barrier cells [185]. Either way, these observations set the frame for a novel non-osmotic, non-hemodynamic stimulation of AVP secretion. In contrast, other ICU-associated stressful conditions do not interfere with water excretion and the sole admission in ICU may not be incriminated *per se* in the occurrence of SIADH and hyponatremia [184]. Finally, elderly patients warrant special attention as old age predisposes to the development of SIADH [190, 191]. More specifically, the latter may only be unveiled when the patients are exposed to high volumes of hypotonic fluids.

##### Exercise-induced hyponatremia

Hyponatremia is common among subjects submitted to strenuous exercises. In one study, 13 % of marathon runners presented with hyponatremia and 0.6 % had severe hyponatremia (120 mmol/L or less) [192]. Weight gain, a plausible reflection of the volume of potential overhydration is a strong predictor of the occurrence of hyponatremia as well as female gender and the duration of exercise [193]. Widely viewed as a consequence of excessive sodium and chloride loss through sweating, hyponatremia, in fact, fulfills the criteria for SIADH [194, 195]. It has been postulated that IL-6, a cytokine released in this case as a consequence of muscle glycogen depletion following prolonged racing, may serve as a trigger for non-osmotic AVP secretion [185, 196].

#### Hyponatremia in the setting of increased ECFV

Three subsets of conditions may be delineated, when confronted with hyponatremia in the context of increased ECFV. Urinary sodium levels (<20 mmol/L), which mirrors secondary hyperaldosteronism and enhanced tubular sodium reabsorption, should prompt a high index of suspicion with regards to heart failure, cirrhosis and nephrotic syndrome [197].

There is a common ground between heart failure, cirrhosis and nephrotic syndrome with respect to the mechanisms implicated in the development of hyponatremia, namely a state of arterial underfilling which results, as the physiopathological scenario unfolds (see “Basic principles of sodium and water equilibrium”), in an increased release of AVP, an augmented adrenergic drive and the stimulation of the plasma renin angiotensin aldosterone system [12, 46, 198–200].

In cirrhosis, as in heart failure, arterial underfilling, and its ensuing consequences, are also the predominant hypothesis behind the physiopathology of hyponatremia [201]. The difference lies in what causes arterial underfilling, namely splanchnic arterial vasodilatation, itself a consequence of increased nitric oxide production in the setting of advanced cirrhosis.

The incidence of hyponatremia is reputed a less common accompaniment of nephrotic syndrome than other oedematous states [27]. Hyponatremia has also been interpreted as the expression of arterial underfilling by virtue of Starling’s principle. More specifically, fluid is thought to translocate from the vascular space to the interstitial space, a phenomenon elicited by the decline in oncotic pressure, thereby lowering the effective arterial blood volume. In line with this, physiological investigations in children with nephrotic syndrome have revealed elevated levels of AVP [202]. However, this theory is undermined by conflicting results regarding the level of effective arterial blood volume in nephrotic patients and the numerous flaws attached to Starling’s principle [203].

#### Hyponatremia and decreased ECFV

First of all, it should be kept in mind that hypovolemia, irrespective of its cause, is a powerful inductor of AVP release so that any concomitant input of hypotonic fluids is bound to generate hyponatremia. In other word, hyponatremia is likely to be both “dilutional” and “depletional” even in the face of overt sodium wasting and these terms should be abandoned.

Broadly speaking, patients can be categorized either as having sodium losses via the kidney (diuretics, adrenal insufficiency, salt-wasting nephropathies and the so-called cerebral salt-wasting syndrome) or through the digestive tract.

Measuring the sodium urine concentration may provide clues to which is the route involved in sodium wasting (see Fig. 2). Typically, when hypovolemia arises from digestive losses, urinary sodium is expected to be low (<20 mmol/L), as a result of secondary hyperaldosteronism. One exception to this rule is hyponatremia related to vomiting. In this case, bicarbonate urinary excretion is increased in order to counteract the state of metabolic alkalosis that results from gastric losses of hydrochloric acid. Renal excretion of sodium ensues as bicarbonaturia requires the concomitant excretion of a cation. Another caveat comes from the usage of diuretics which will increase the urinary sodium concentration rendering its interpretation challenging.

Thiazide diuretics have carved out a preponderant position in the therapeutic arsenal against arterial hypertension thanks to clinical trials which have established their efficacy [204]. Along with their popularity thiazides have also unfortunately rose to become the leading cause of drug-induced hyponatremia [205]. Thiazide diuretics primarily act by inhibiting the transport of sodium and chloride in the distal convoluted duct and connecting segment. This impedes the lowering of the tubular fluid osmolarity to its minimum value and, consequently, reduces electrolyte-free water excretion [206, 207]. Besides hampering the tubules diluting capacity, thiazides also induce potassium depletion, another factor propitious to the development of hyponatremia, given that PNa levels are correlated to the total exchangeable potassium [10]. In other words, a contraction of the potassium pool is per se sufficient to explain the occurrence of hyponatremia, in agreement with Edelman’s equation. Finally, thiazide-induced volume depletion is mild and, in any case, delayed, so that it is not likely to be a major player in the pathogenesis of hyponatremia. At variance, loop diuretics represent a relatively infrequent cause of hyponatremia [208]. Their action is short lived allowing for repletion of sodium stores. Most of all, loop diuretics alter concentration abilities as well to dilution abilities, in contrast to thiazide diuretics which only affect the latter [207].

If hyponatremia does not reliably predict relative adrenal insufficiency in critically ill patients [209], it is a salient feature of primary adrenal insufficiency [210, 211]. In this context, hyponatremia stems from both the loss of glucocorticoid and mineralocorticoid functions. However, hypovolemia reflects the mineralocorticoid deficiency which manifests itself by depressed levels of aldosterone and ensuing urinary sodium wasting. In these conditions, water will be maximally retained and hyponatremia will ensue.

Cerebral salt-wasting syndrome (CSW) remains at this time a controversial entity and its very existence has fuelled debate, some authors considering this entity as a misnomer for SIADH [212]. To qualify for salt-wasting syndrome, patients must display signs of reduced extracellular volume along with inappropriate sodium losses [173, 213] in the presence of a cerebral insult, predominantly subarachnoid hemorrhage [214]. However, an inadequately elevated natriuresis and a context of a central nervous lesion are not distinctive of SIADH, so that to differentiate CSW from SIADH one has to rely on the assessment of extracellular volume, a difficult task in routine practice as previously outlined. Regarding its biological characterization, yet another caveat lies in the fact that reduced uric acid levels may be found in patients with presumed CSW, similar to SIADH, perhaps as a consequence of defective tubular reabsorption of uric acid [213, 215]. Nevertheless, studies resorting to isotopic determination of extracellular volume have hypothesized that CSW might be more prevalent in the setting of neurosurgical ICU than is SIADH [216, 217]. The pathogenesis of CSW remains elusive. Some have surmised that CNS damage caused a breakdown in the sympathetic-mediated stimulation of renin secretion by the juxtaglomerular cells, thereby leading to diminished levels of aldosterone and a reduction of sodium reabsorption in the collecting duct [218, 219]. One major shortcoming associated with this theory is that a surge in the adrenergic tone, rather than a decrease in sympathetic activity, has been reported in acute CNS disease or injury. In fact, this serves as a basis for an alternative physiopathological hypothesis to CSW; the increase in sympathetic activity may stimulate the secretion of brain natriuretic peptide (BNP) secretion by the myocardial tissue [220]. In conclusion, this diagnosis must be envisaged with caution. Its distinction with SIADH remains elusive and, in any case, hypertonic saline solution administration under strict monitoring is mandatory [212], together with tight hemodynamic and biological monitoring (PNa, natriuresis, renal function).

Salt-wasting nephropathies encompass multiple heterogeneous and unrelated kidney diseases, most of which are anecdotal causes of hyponatremia. They include congenital obstructive uropathy [221], cisplatin-induced nephropathy [222], interstitial nephritis [223], medullary cyst disease [224] and salt-wasting tubulopathies (congenital or acquired type I pseudo-hypoaldosteronism [225], Bartter and Gitelman syndromes).

## The management of hyponatremia

### The principles of PNa correction in case of severe hyponatremia and hyponatremic encephalopathy

Profound hyponatremia with concurrent severe neurological symptoms is a medical emergency and warrants prompt therapy. The pivotal treatment is hypertonic saline (3 % NaCl, 513 mmol/L) delivered intravenously. 0.9 % NaCl is not an appropriate solution as its tonicity is insufficient to ensure an adequate raise of PNa levels. Volume overload is an obvious contraindication to hypertonic saline therapy, but the great bulk of patients managed for profound, symptomatic hyponatremia usually display normal or diminished ECFV. Administration through a nasogastric tube should be avoided because enteral absorption is impaired and unpredictable in critically ill patients. In contrast, both continuous intravenous and bolus therapy are acceptable administration modalities. The optimal magnitude of increase in PNa is hitherto unknown, varies according to experts and proposed aims, does not rely on sound evidence or is based on studies in other settings [226]. For instance, in the context of brain injury, a 4 mmol/L increase in PNa levels resulted in significantly diminished intracranial pressure [227]. In a retrospective assessment of 60 children suffering from hyponatremic seizures, investigators concluded that a 3–5 mmol/L rise in PNa was enough to terminate seizure [228]. Consistently, recent guidelines have advocated a rapid 4–6 mmol/L increase in PNa levels (which equates to an increase of 8–12 mmol/L in effective osmolality) as a reasonable target [70, 229, 230]. Likewise, the rate of PNa correction rate has fuelled much debate [231] and it does not lend itself well to straightforward computation [230]. Nonetheless, a consensus for a rapid, albeit time-limited rise in PNa has surfaced. In agreement with this concept, a mean correction rate of 1.3 mmol/L/h resulted in satisfactory outcome among 33 patients, among which 12 presented with coma [232]. Therefore a correction rate of 1.5–2 mmol/L/h appears to be safe and effective, provided it is restricted to the first 3–4 h following the initiation of the hypertonic saline therapy [229]. As a rule of thumb, a single bolus of 2 cc/kg of 3 % NaCl yields an increase of PNa approaching (but inferior) to 2 mmol/L [226]. More sophisticated formulae have been devised to predict the increase in PNa following sodium repletion, one of the most popular being the so-called Adrogue–Madias equation [113]. Great caution should be exercised when resorting to these computations as they have been found to underestimate the true magnitude of PNa increase [233]. Notably, the Adrogue–Madias formula relies on the gross assumption that the total body water content is constant and discards ongoing sodium, potassium and water losses as well as spontaneous correction [234]. In any case, a close monitoring of PNa levels is mandatory and should be repeated every 2 h.

Loop diuretics which promote enhanced electrolyte-free water excretion may be useful, especially in the setting of coexistent fluid overload and in SIADH. Notably, one study established that urine sodium averaged 60 mmol/L (±47 mmol/L) after furosemide therapy indicated for heart failure [235]. With respect to SIADH, this condition forms the only acceptable reason to concurrently administer sodium (which will allow for a brisk increase PNa levels) and diuretics (that help eliminating free water, thus bolstering the rise in PNa levels).

Finally, a meticulous scrutiny for evidence of hypoxia and seizures is mandatory as both may aggravate cerebral edema. Practitioners should resort liberally to mechanical ventilation in case of any one of these complications occurs yet the utmost care should be taken when performing endotracheal intubation to avoid hypoxemia during this procedure. At variance, anticonvulsant therapy is seldom efficacious [228].

Exercise-associated hyponatremia has been the object of guidelines [236]. Intravenous normal saline (0.9 % NaCl, 154 mmol/L Na+) administration runs the risk of further aggravating hyponatremia given that AVP levels are bound to be very elevated in this setting. Thus only athletes exhibiting clear signs of decreased ECFV are likely candidates for this therapy [193, 237]. Hypertonic 3 % saline therapy through intravenous administration is the safest option whenever participants exhibit profound hyponatremia and/or neurological symptoms [236–238]. Experts have advocated the onsite administration of 100 mL of 3 % NaCl over 10 min [236]. Finally, concurrent pulmonary edema (putatively neurogenic) should not deter physicians to administer 3 % hypertonic saline as this treatment can be life-saving in such circumstances [238, 239].

If decreased ECFV is the prime mechanism underpinning hyponatremia, then intravenous normal saline therapy is the most judicious choice, besides addressing the cause responsible for the contracted EFCV. Of note, normal saline therapy, routinely deemed as isotonic is in fact hypertonic relatively to the plasma osmolarity of hyponatremic patients.

Whenever adrenal failure is suspected to be the culprit behind the reduced ECVF, administration of 100 mg of hydrocortisone should be emergently administrated to the patient, after blood sampling for cortisol assessment, followed by 100–200 mg of hydrocortisone per 24 h. Fludrocortisone is however unnecessary in the acute setting given the mineralocorticoid potency of hydrocortisone when used at high doses [240]. The cornerstone for treating patients with suspected CSW is sodium replacement and the restoration of a normal extracellular volume. 0.9 % NaCl is a reasonable first choice treatment but physicians may switch to hypertonic saline therapy (either 1.5 or 3 % NaCl) in case PNa levels fail to increase [214].

### Dodging the risk of OD

#### Settings at risk for PNa overcorrection

At this point, one reservation should be made about the classic differentiation between chronic and acute hyponatremia, in terms of risk of OD. Although there is undeniable evidence (as exposed previously) that patients with chronic hyponatremia face a higher risk of OD, from a clinical standpoint this distinction is questionable, as the exact onset of hyponatremia is seldom readily available [241]. One of the better means to prevent OD is to limit the occurrence of hyponatremia. In the ICU setting special attention should be paid to ill-motivated prescription of hypotonic fluids, especially in the post-operative setting.

The next step is to recognize conditions propitious to the overcorrection of hyponatremia. A profuse electrolyte-free diuresis accompanied with a brisk rise in PNa levels is likely to be observed in case of primary polydipsia (after implementing water restriction), impaired dilution (after discontinuation of the culprit drugs, thiazide diuretics or anti-psychotic drugs), or after restoration of a normal diluting ability once a decreased ECFV has been replenished.

When hyponatremic patients are placed on renal replacement therapy exceedingly high correction of PNa levels may be thwarted by reducing the sodium concentration in the dialysate and shortening the dialysis time.

#### Setting PNa target levels

The optimal PNa target levels remain an area of controversy. Expert panel recommendations have consistently revised PNa variation to lower thresholds, based on accumulating evidence that even small variations of PNa levels may expose hyponatremic patients to the risk of OD. Originally, 24- and 48-h maximally tolerated PNa increases were set at 12 and 25 mmol/L, respectively [78]. Currently, there is a broad consensus that the 48-h PNa limit should in fact not exceed 15–20 mmol/L. As for the 24-h variation limit, goals differ considerably with levels ranging from 4 to 10 mmol/L, according to various experts [67, 68, 113]. Advocates of the 4–6 mmol/L maximum 24-h correction contend that a 4 mmol/L rise in PNa levels is sufficient to improve the patient’s neurological condition, as previously discussed, and that OD is arguably unlikely to develop when the rise in PNa levels is confined below these limits. The off-side is that keeping PNa levels within such narrow margins requires considerable expertise and, at any rate, very cautious monitoring. Another challenge arises from the fact that maintaining a steady and limited PNa augmentation should be carried out until PNa correction has been fully achieved i.e. the 24- and 48-h PNa variation limits should not be misinterpreted as requirements restricted to the initial first 24 and 48 h, as overcorrection can occur at any time point. Finally, a new concept is gradually emerging: rather than rigid, universally-applied PNa thresholds, PNa correction rates should be tailored individually, depending on each patient’s risk factors for OD [67]. Figure 2 summarizes the goals of PNa correction.

#### Dealing with PNa overcorrection

**Fig. 3:**
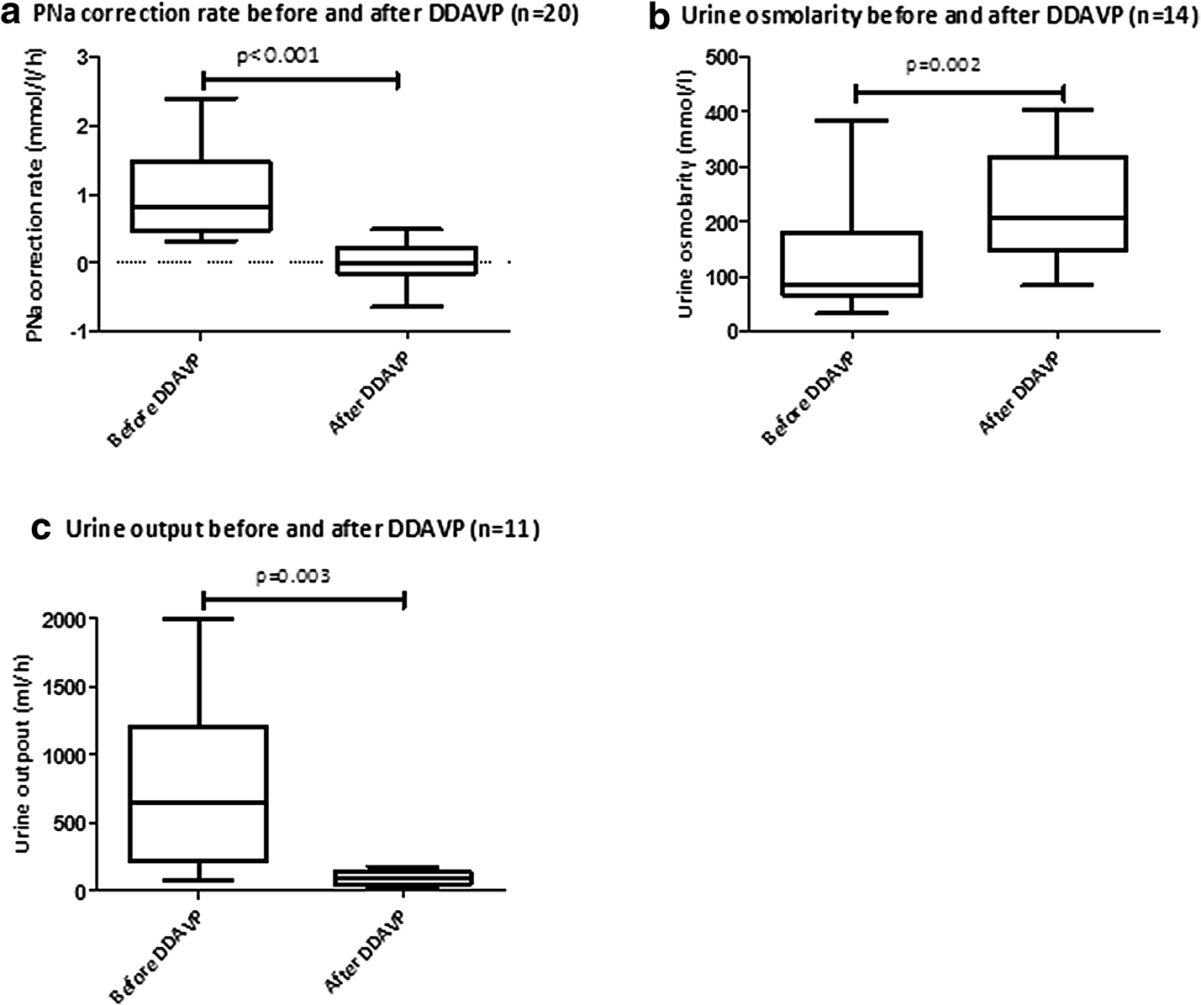
Effects of DDAVP on PNa kinetics and urine composition and output in 20 patients admitted in the ICU for severe or profound hyponatremia: **a** PNa correction rate before and after DDAVP administration (*n* = 20). **b** Urine osmolarity before and after DDAVP administration (*n* = 14). **c** Urine output before and after DDAVP administration (*n* = 11). The *box plots* indicate median, interquartile ranges (25th and 75th percentiles), and minimum and maximum values. DDAVP administration allows for a marked reduction in electrolyte-free water output along with a significant reduction in the PNa correction rate. Reproduced with permission of the *Clinical Journal of the American Society of Nephrology* [246]

**Fig. 4:**
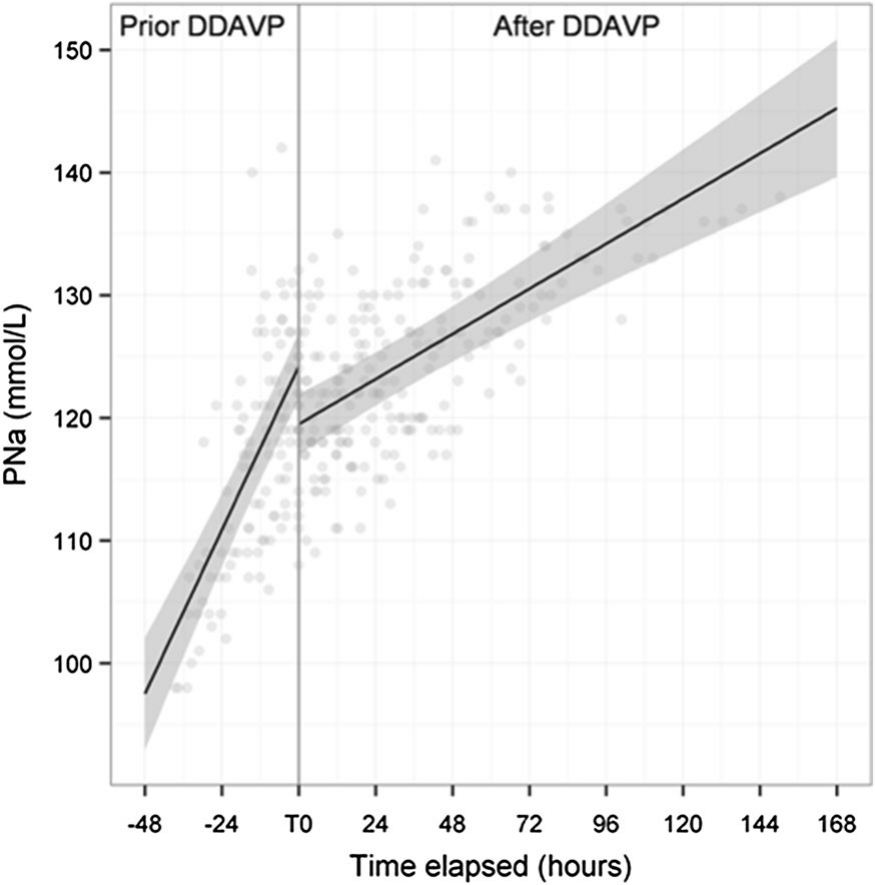
Rates of plasma sodium concentration increase before and after DDAVP administration output in 20 patients admitted in the ICU for severely symptomatic or profound hyponatremia. The slope of PNa was significantly reduced from 0.5860.12 to 0.1560.043 mmol/L/h (*p*, 0.001) after DDAVP administration. DDAVP administration thus likely avoided PNa overcorrection. Reproduced with permission of the *Clinical Journal of the American Society of Nephrology* [246]

In the event of PNa overcorrection, several studies have suggested that halting PNa levels increase and even re-lowering PNa levels may be protective. In rats subjected sequentially to hyponatremia followed by a sudden restoration of normal PNa levels, re-inducing a mild level of hyponatremia reduced neurological manifestations and decreased mortality, as compared to a control group of rats deprived of such therapy [242, 243]. In the clinical field, anecdotal evidence supports PNa re-lowering as a means to offset OD; it consists in few case reports which have depicted the reversal of OD following PNa re-lowering [244, 245]. Nonetheless, given the calamitous prognosis of OD most expert recommendations argue in favor of this strategy [67, 68]. One hindrance to PNa re-lowering is that it is usually achieved at the expense of the repeated infusion of large volume of hypotonic fluids. Not only is it time-consuming for caregivers but it may also prove ineffective. An alternative resides in the administration of desmopressin, a V_2_-vasopressin analogue, which promotes water reabsorption through V_2_ receptors activation on the collecting duct, thereby reducing urinary water losses which serve to increase the PNa. It bears the advantage of stopping immediately any further increase of PNa levels. PNa levels can thereafter be easily manipulated by either administering moderate amounts of hypotonic fluids (i.e. dextrose 5 % in water or even dextrose 2.5 % in water so as to avoid hyperglycemia) either to re-lower PNa to safer levels or to stabilize PNA levels. This technique has proven to be safe and effective in terms of PNa kinetics in three independent studies, and some investigators have even suggested it may be used in combination with 3 % NaCl [246–248]. In a retrospective study involving 20 patients admitted in two ICU for hyponatremia defined as PNa levels inferior to 120 mmol/L and/or neurological symptoms in conjunction with an excessive rate of PNa increase, the use of DDAVP dramatically reduced the rate of PNa correction from a median 0.81 mmol/L/h [interquartile range 0.46, 1.48] to −0.02 mmol/L/h [−0.16, 1.48] (*p* < 0.001) along with a concurrent decrease in urine output (650 mL/h [214, 1200] versus 93.5 mL/h [43, 143]; *p* = 0.003) and a rise in urine osmolarity (86 mmol/L [66, 180] versus 209 mmol/L [149;318]; *p* = 0.002). Strikingly, the magnitude of PNa variation were also significantly dampened with a maximum variation of 11.5 mmol/L [8.25, 14.5] versus 5 mmol/L [4, 6.75] (*p* < 0.01) before and after DDAVP, respectively [246] (see Figs. 3, 4).

There is currently no effective treatment for OD but experimental models have provided new insights on its physiopathology. More specifically, glucocorticoids (by restoring the blood–brain barrier and its anti-inflammatory properties) [249] and myo-inositol (by repleting brain cells deficiencies in organic osmoles) [250, 251] might represent avenues for new therapies in the future.

### Treating hyponatremia in specific settings

Physicians should restrain from an overly aggressive correction of chronic hyponatremia. The goal is rather to obtain a slow, gradual correction of PNa levels since the risk for hyponatremic encephalopathy is limited and outweighed by the likelihood of OD [229]. In most instances, this can be achieved by discontinuing potential offending drugs (see Table 3) and/or implementing an oral fluid restriction protocol.

In case of volume overload, the mainstay of patient management is water restriction and loop diuretics. Aquaretics (vaptans, see below) require further investigation before they can be regarded as standard of care [252].

The first-line treatment is water restriction in case of mild, well-tolerated hyponatremia subsequent to SIADH. The response to water restriction is expected to be favorable when urine osmolality is relatively low (<400 mOsm/H_2_O/kg), but even in such case a rise in PNa levels is delayed until a couple of days after its implementation. Otherwise, loop diuretics (together with salt repletion) and vaptans should be the privileged therapeutic options [174].

### Pharmacological therapy of hyponatremia in the ICU: old drugs, novel therapies and future perspectives

Various pharmacological strategies have been devised to accomplish one common goal which is to reset sufficient levels of urine osmolarity in order to obtain a positive free water clearance. Drugs achieve this either by increasing the osmolar load or restoring tubular diluting capacity.

Urea is a small diffusible molecule that is filtered unrestrictedly by the glomerulus and generates an obligatory osmotic diuresis when given at high doses. In the setting of SIADH, urea has been proven to be a cost-effective means to achieve a gradual improvement of PNa level. More recently, investigators have shown similar results in critically ill patients, however the underlying mechanism of hyponatremia was predominantly SIADH and patients displayed mild to moderate hyponatremia (mean PNa = 124.8 mmol/L) [253, 254]. Due to its delayed effect on PNa levels, this therapy may not be contemplated when managing patients with profound and symptomatic hyponatremia. Furthermore, patient compliance to this drug is hampered by its displeasing taste [168].

Demeclocycline, an antibiotic, which belongs to the tetracycline family, counteracts the effects of AVP by inducing a partial, reversible nephrogenic diabetes insipidus [255, 256]. Although studies have ascertained its effectiveness in controlling euvolemic hyponatremia (SIADH), its use in practice is hampered by its nephrotoxicity [257].

Vaptans have emerged recently as promising drugs in the treatment of euvolemic and hypervolemic hyponatremia. They act as competitive antagonists to AVP hindering its binding to its receptors (mainly V_2_ receptors) located on the basolateral pole of the collecting ducts. The result is a down regulation of AQP2 and an increase of electrolyte-free diuresis [258]. Taken as a whole, studies focusing on patients with hyponatremia related to congestive heart failure [259, 260], cirrhosis [259, 261] and SIADH [259] have documented a positive effect of the drug on PNa levels but have failed to demonstrate that the reversal of mild to moderate hyponatremia equates with an improvement in meaningful outcomes. Furthermore, hyponatremic patients do not respond uniformly to vaptans, cirrhotic patients being less responsive to their effects [262]. Numerous impediments hamper the use of vaptans in the ICU setting. Their action is not immediate and they provide only for a modest increase in PNa levels which makes vaptans unsuitable for the treatment of highly symptomatic hyponatremia, unless they are used in combination with other strategies [263, 264]. To date, only conivaptan may be delivered intravenously and its use in the ICU has been confined to case reports or small non randomized studies [265]. After a loading dose, administration through a continuous intravenous perfusion has been advocated. However, this regimen comes with a greater risk of vascular irritation and in-site thrombosis [266]. Other notable side effects include hypokalemia, headaches, thirst and drug interactions due to competition between vaptans and other CYP3A4 substrates. Finally, although seemingly rare, the risk of PNa overcorrection does exist and special attention should be paid regarding the occurrence of hyperaquaresis upon treatment initiation, especially in cases of chronic SIADH.

How vaptans will fit in the therapeutic armamentarium of severe symptomatic hyponatremia is thus uncertain. Whether vaptans will be utilized as an adjuvant therapy to reinforce other measures, or as part of a sequential strategy as a relay treatment to hypertonic saline is left to speculation [267]. Finally, one must keep in mind that vaptans are expensive drugs.

## Conclusion

Hyponatremia remains a major bane for the intensivist. Not only is it the most frequently encountered electrolyte disorder, but it is increasingly recognized as a harbinger of dismal outcome in various conditions. The appropriate management of hyponatremia requires its timely recognition, a judicious analysis of the underlying mechanisms involved in its pathogenesis, and the appreciation of its severity and impact on the patient’s course. Above all, hyponatremia poses a conundrum which resembles the likes of a Zugzwang situation in chess when any move worsens the player’s position. In a similar vein, attending physicians should be reminded that when confronted with profound and symptomatic hyponatremia, overtreating a patient may be as deleterious as failing to initiate the right therapeutic measures. Fortunately, the understanding of the physiopathology of both hyponatremic encephalopathy and OD has achieved considerable success and may give rise to novel therapies in the future.

## Abbreviations

ICU: intensive care unit
OD: osmotic demyelination syndrome
PNa: plasma sodium concentration
AVP: arginine vasopressin
CNS: central nervous system
OVLT: organum vasculosum laminae terminalis
SON: supraoptic nuclei
TRP: transient receptor potential
PVN: paraventricular nuclei
AQP: aquaporin
ENac: epithelial sodium channel
ECFV: extracellular fluid volume status
SIADH: syndrome of inappropriate antidiuretic secretion
CSW: cerebral salt wasting syndrome
